# Highly sensitive and quick in ovo sexing of domestic chicken eggs by two-wavelength fluorescence spectroscopy

**DOI:** 10.1007/s00216-022-04446-0

**Published:** 2022-12-03

**Authors:** Grit Preuße, Vincenz Porstmann, Thomas Bartels, Christian Schnabel, Roberta Galli, Edmund Koch, Martin Oelschlägel, Ortrud Uckermann, Gerald Steiner

**Affiliations:** 1 Department of Anesthesiology and Intensive Care Medicine, Clinical Sensoring and Monitoring, Faculty of Medicine Carl Gustav Carus, Technische Universität Dresden, Fetscherstr. 74, 01307 Dresden, Germany; 2 Institute for Animal Welfare and Animal Husbandry, Federal Research Institute for Animal Health, Friedrich-Loeffler-Institut, Dörnbergstr. 25/27, 29223 Celle, Germany; 3 Department of Medical Physics and Biomedical Engineering, Faculty of Medicine Carl Gustav Carus, Technische Universität Dresden, Fetscherstr. 74, 01307 Dresden, Germany; 4 Division of Medical Biology, Department of Psychiatry and Psychotherapy, Faculty of Medicine Carl Gustav Carus, Technische Universität Dresden, Fetscherstr. 74, 01307 Dresden, Germany

**Keywords:** In ovo sexing, Fluorescence spectroscopy, Two-wavelength spectroscopy, Heme synthesis, Protoporphyrin IX

## Abstract

The in ovo sexing of chicken eggs is a current task and a prerequisite to overcome the mass killing of male day-old chicks from laying lines. Although various methods have been developed and tested in recent years, practicable methods for sex determination are still missing which can be applicated in poultry hatcheries before the chicken embryo is capable of nociception and pain sensation. Optical spectroscopic methods enable an early determination of the sex. In this study, a novel method based on two-wavelength in ovo fluorescence excitation is described. More than 1600 eggs were examined. In ovo fluorescence was sequentially excited at 532 nm and 785 nm. The fluorescence intensities of the spectral regions behave inversely with respect to sex. It is shown that the observed sex-related differences in the fluorescence intensities are based on the embryonic hemoglobin synthesis. The accuracy of sex determination is 96% for both sexes. The hatching rate is not reduced compared to an equivalent reference group.

**Graphical Abstract:**

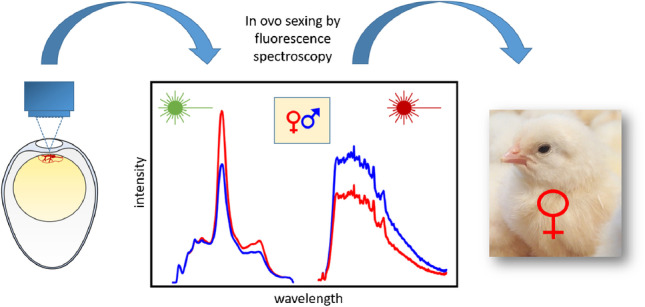

## Introduction

Farm chicks are bred for two purposes: to lay eggs or to produce meat. Male chicks of layer lines are divided from their sisters within a day of hatching and culled, which is one of the biggest animal-welfare issues in the egg production [1]. In many states, egg producers are being pressured to phase out chick culling. For example, the United Egg Producers stated to stop the mass culling of male chicks as soon as there is an ethical and economically feasible alternative [2]. In Germany, the culling of hatched male day-old chicks has been banned by law since 2022, and from 2024 the culling of chicken embryos has to be done at an early state but not later than day 7 of incubation [3].

Therefore, the early sexing of fertilized eggs is considered as the key to overwhelm the culling of unwanted male day-old chicks. Consequently, the in ovo sexing of chicken eggs has been a world-wide research interest since over a decade [1]. Several groups have investigated techniques for an in ovo sexing by analyzing individual marker molecules or sum parameters like the biochemical profile of embryos. Another strategy to avoid the killing of hatched male chicks is based on CRISPR/Cas 9 gene editing in parent flocks [4, 5]. A green fluorescent protein gene is micro-injected in the embryo at the parental level and place on the female Z chromosome. At production level, homozygotic male embryos (ZZ) can be identified due to their fluorescence and eggs can be sorted out. In the female embryos, the unchanged Z and W chromosomes are active. Laying hens that develop from these embryos as well as the eggs they lay are not genetically modified. However, methods that allow the identification of female and male embryos by means of genetic engineering are publicly criticized and will probably not be used due to a lack of consumer acceptance, at least in Western European countries [6].

Table 1 summarizes the current bioanalytical methods for in ovo sexing of hatching fertilized eggs. It should be noted that occasional also not reproducible studies of sexing eggs were published, for examples by morphological measurements [7] of the egg, which are not considered in Table 1.

**Table 1:** Known scientific methods for sexing hen’s eggs. (Abbreviations of the domestic chicken breeding line: *LB* Lohmann Brown, *LSL* Lohmann Selected Leghorn)

Method	Applicable at day	Accuracy	Time for determination	Hatching rate	Remarks	Reference
Terahertz spectroscopy (‘TeraEgg’ technology)	1	Not specified	Approx. 20 min	Not specified	Small-scale test	[8]
Solid phase microextraction- gas chromatography – mass spectrometry	≥ 1	Not specified	Several minutes	Not specified	Analysis of volatile organic compounds	[9]
Genome editing (‘eggXYT’ technology; ‘CSIRO’ technology)	2.5	Not specified	Not specified	Not specified	CRISPR technology	[4, 10]
Raman spectroscopy	3.5	Better 90%	20 s	Not reduced	No chemicals, needs to open the egg	[11–13]
Fluorescence spectroscopy	3.5	Better 93%	5 s	Not reduced	No chemicals, needs to open the egg	[12–14]
Endocrinology (‘Seleggt’ technology)	≥ 9	84% day 9 99% day 10 [LB] 100% day 10 [LSL]	Not specified	90.1% [LB] 81.3% [LSL]	Sampling through a small hole, needs test kit	[15, 16]
DNA-PCR-analysis (‘Plantegg’ technology)	≥ 9	99.5%	3600 eggs per hour and device	Not specified	Sampling through a small, needs PCR kit	[17]
Mass spectrometry (‘Ella’ technology)	≥ 9	Not specified	< 1 s	Not specified		[18]
Vis-optical Imaging (‘Cheggy’ technology)	≥ 13	> 96%	Not specified (20,000 eggs/h)	92.5%	Only suitable for lines with sex-related down feather color	[19–21]
Visible-near-infrared point spectroscopy	≥ 14	97.78 …99.52%	500 ms …3000 ms	Not specified	Only suitable for lines with sex-related down feather color	[22]
Magnetic resonance imaging (‘Orbem genus’ technology)	≥ 14	Near 100%	900 eggs/hour	Not specified		[23]

**Fig. 1:**
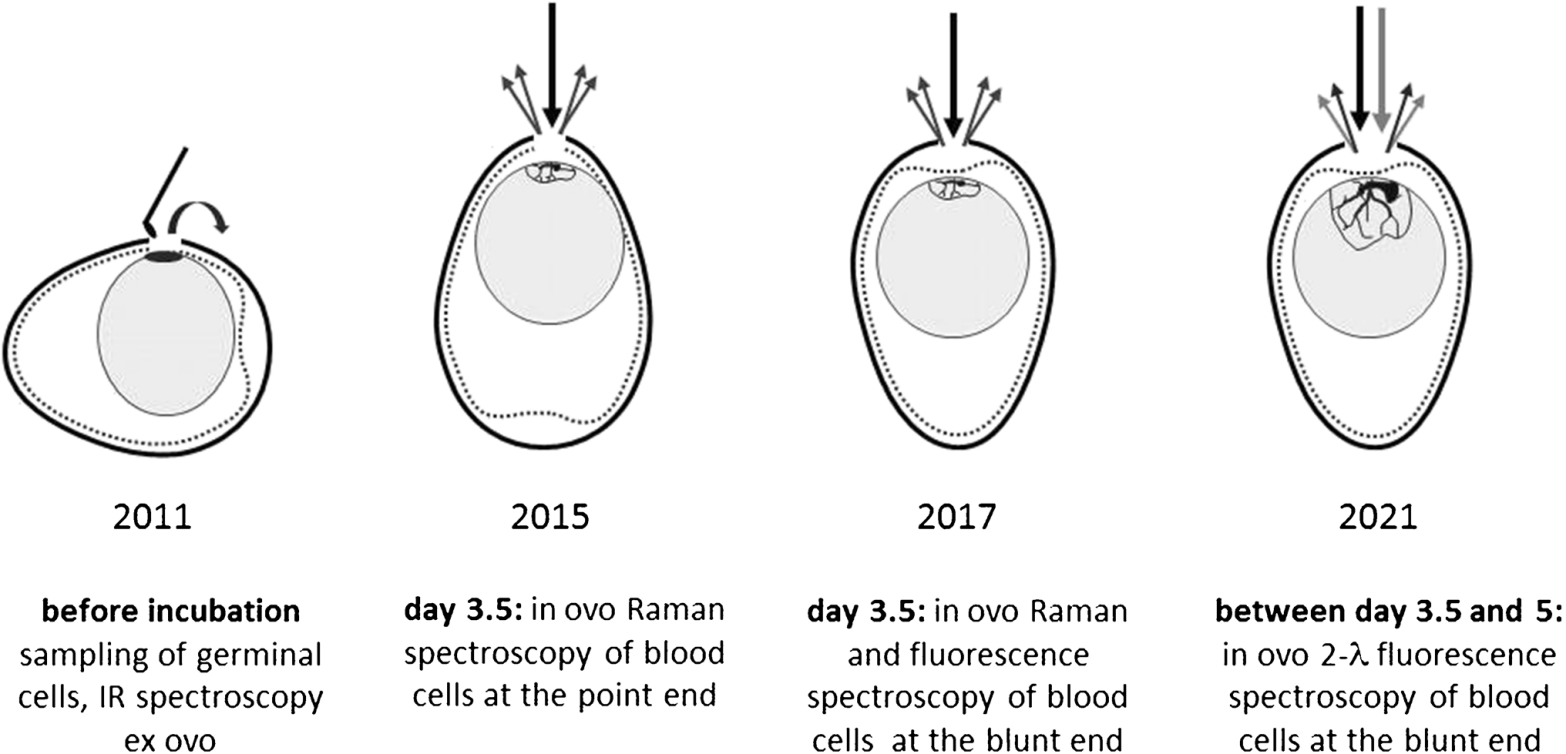
History of spectroscopic methods for the early in ovo sexing of hen’s eggs

Exactly when the ability to nociception begins is not known with certainty. Currently, it is scientifically accepted that the chicken embryo is not yet capable of nociception before the 7^th^ day of incubation. Techniques based on in ovo determination of feather color [19–22] are solely applicable in the late incubation phase and only for brown layers. The endocrinological method is already used commercially, but sex determination cannot be made before the 9^th^ day of incubation [15, 16]. Molecular optical spectroscopic methods [11–14] have the advantage of being applicable in a very early state of incubation. Therefore, these methods will meet the concern of animal welfare and are considered as favorable for a broad practical use in large-scale hatcheries. During the past years, several studies were published about in ovo sexing by Raman and fluorescence spectroscopy. The history of hen’s egg sexing by molecular spectroscopic methods is illustrated and summarized in Fig. 1.

One of the earliest works about sexing eggs by infrared (IR) spectroscopy was published in 2011 [24]. The ex ovo analysis of germinal cells provided satisfactory results in regard to the accuracy but a very low hatching success. Five years later, a new in ovo approach based on the analysis of embryo’s blood cells was published [11]. Raman spectra were recorded from an opened egg without taking samples. The accuracy was around 92%. A small laboratory study of 59 eggs revealed that normal developed chicks hatch from previously Raman-sexed eggs [11]. However, due to the egg opening at the point end, the embryo is extensively unprotected which makes the whole incubation process in hatcheries difficult. In 2017, it was demonstrated that a spectroscopic in ovo sexing is also possible by opening the egg at the blunt end [12, 13]. Thereby, the embryo is protected by the intact inner eggshell membrane. To ensure a high accuracy, embryonic blood vessels have to be focused precisely and the focus has to be kept during the measurement of approx. 20 s. Light scattering and fluorescence effects due to the eggshell membrane have to be compensated resulting in an accuracy of nearly 95% [14]. An internal unpublished study with nearly 250 eggs, performed and documented at the German Federal Research Institute for Animal Health (Friedrich-Loeffler-Institute—FLI, Celle, Germany), has also revealed that the hatching rate is similar to those of untouched eggs.

In this manuscript, we report about a new approach using two different excitation wavelengths to determine molecules of the heme synthesis pathway. This concept makes the early spectroscopic in ovo sexing more precise, robust, quick, and also practicable for an industrial use. The study of spectroscopic in ovo sexing described here also shows that analytical chemistry contributes to solving one of the biggest problems of animal welfare. At present, the spectroscopic method appears to be the only method for in ovo sexing that can meet both the operational requirements of hatcheries and the ethical principles or legal requirements of animal welfare.

## Materials and methods

### Egg handling

Incubation of eggs, spectroscopic in ovo sexing, and hatching of the chicks have been performed at the FLI. Fertilized eggs of a white layer strain (LSL) were used, obtained from Lohmann Breeders GmbH (Cuxhaven, Germany). Immediately after delivery, the eggs were inspected and stored at approx. 16 °C for 2 to 4 days until starting incubation. Damaged eggs were discarded and not incubated. The incubation was performed in an automatic egg incubator (P96 vision, Petersime, Zulte, Belgium) at 37.8 °C (100 F) and a humidity of 84%, in vertical position with the pointed end downward upon applying a ± 45° tilt at 1 h cycle.

Eggs were incubated until approximately day 4. In a time period between 93 and 106 h of incubation, the eggs were taken out of the incubator and numbered. Eggshells were windowed in the air cell at the blunt end using a CO_2_ laser (Firestar v30, Synrad, Mukilteo, Washington, USA) equipped with a scanning head (FH Flyer, Synrad, Mukilteo, Washington, USA). The shells were scribed along a circle of 12.8-mm diameter. The shell window was manually opened with a scalpel in order to gain optical access to the embryonic blood vessels. The opening of the egg was performed immediately before the spectroscopic in ovo sexing. The inner shell membrane was not injured. Unfertilized eggs or eggs with an embryo that has died during incubation were sorted out.

After the spectroscopic in ovo sexing, the shell windows were closed using a biocompatible adhesive tape (Leukosilk, BNS Medical GmbH, Hamburg, Germany) and further incubated to hatch on day 21. In total, 1641 in ovo sexed eggs and 1749 untouched eggs as control group were finally incubated. Between days 17 and 18, the eggs were candled in order to count and discard eggs with dead-in-shell embryos. All eggs with well-developed embryos were transferred to a hatcher, where temperature and humidity were changed to 37.3 °C (99.1F) and to 95%, respectively. In the hatcher, eggs were placed into modified trays providing individual hatching boxes to ensure a precise assignment of in ovo sexing results to hatched chicks. Reference sexing of 1581 hatched chicks was done by the standard visual feather sexing protocol as described elsewhere [25].

### Optical layout of the measuring head

**Fig. 2:**
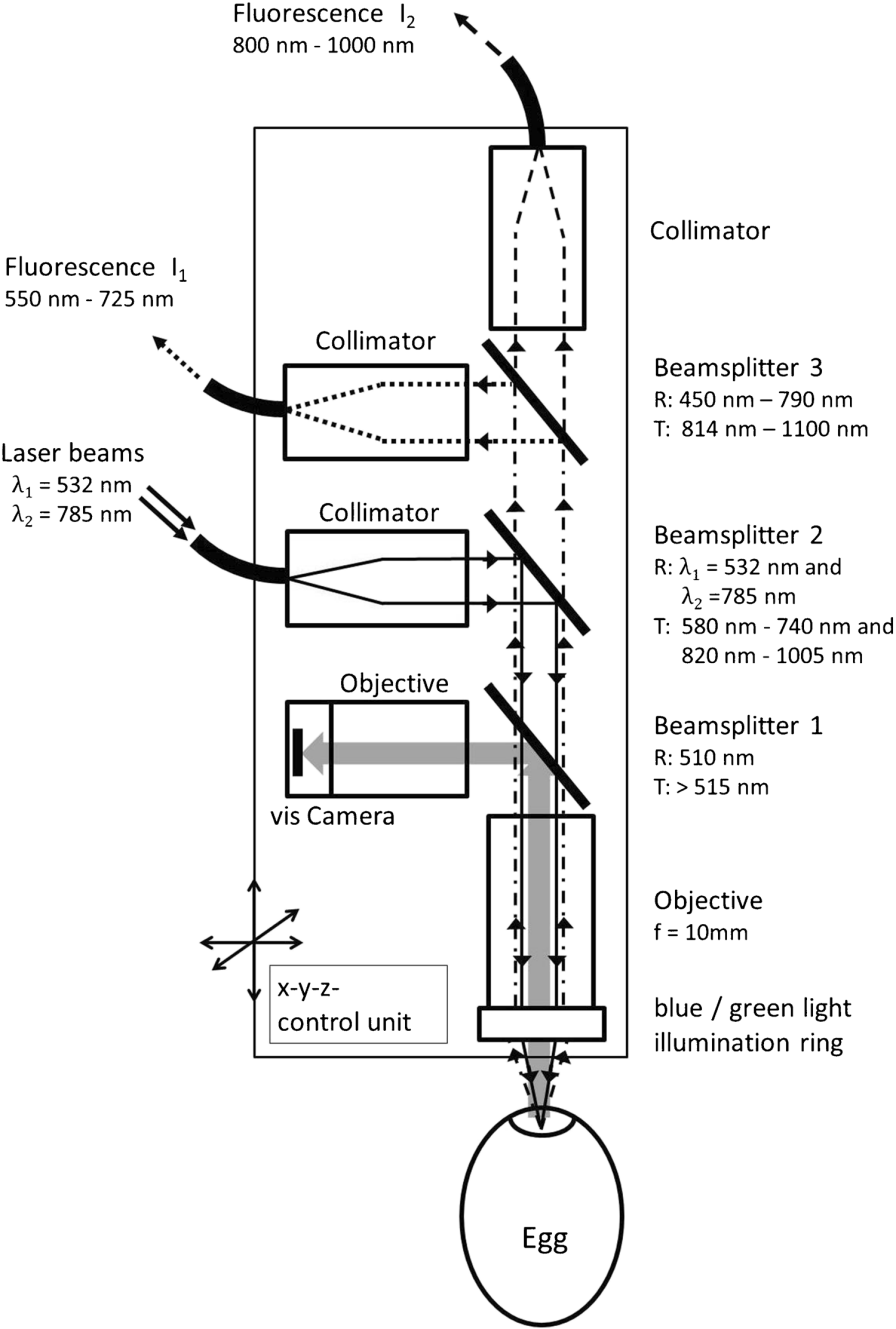
Schematic layout of the in ovo spectroscopic measurement head **(**R, reflection; T, transmission)

For the spectroscopic in ovo sexing, a measuring head was developed that allows fluorescence excitation with two different wavelengths and registration of fluorescence in two separate spectral regions. The entire optical system of the measuring head was built in-house using commercially available components. Figure 2 schematically shows the optical layout of the measuring head.

All optical components are rigidly connected to a motorized unit. The whole unit is computer controlled and can be positioned in three spatial directions so that an optimal in ovo measuring point can be selected and kept in position. Excitation lights and fluorescence signals are guided via multimode fibers. The excitation is provided by two laser sources, a diode pumped solid state laser and a diode laser, both with maximum power of 500 mW emitting at λ_1_ = 532 nm and λ_2_ = 785 nm, respectively. The laser beams are coupled into a multimode fiber with a core diameter of 62.5 µm (Thorlabs GmbH, Munich, Germany) and then combined using a wavelength division multiplexer (Optics Limited, Ottawa, Canada). Objectives and beam splitters were purchased from Thorlabs GmbH (Munich, Germany) and Laseroptik GmbH (Gerben, Germany). The beam splitters sequentially separate blue-green light for visual inspection and selection of the in ovo measurement point, the laser excitation lines, and finally the separation of short-wavelength and long-wavelength fluorescence light. Both laser beams are collimated and coupled into multimode fibers. Spectral analysis of short-wavelength fluorescence is performed by an UV–VIS spectrometer FLAME-S-UV–VIS-ES (Ocean Optics B.V, Ostfildern, Germany) in the range 550 to 725 nm. Spectral analysis of long-wavelength fluorescence is performed by a Raman spectrometer QEPRO-Raman (Ocean Optics B.V, Ostfildern, Germany) in the range 800 to 1000 nm.

### In ovo fluorescence spectroscopy

For the quantitative comparability of fluorescence intensities, calibration was carried out by using a cyclohexane standard solution at the beginning of each measurement series.

For selection of an in ovo measuring point, the visible brightfield image of the opened egg was captured by the blue-green light illumination and the vis camera. Based on the brightfield image, an extraembryonic blood vessel with a diameter larger than 40 μm was selected for the registration of the in ovo fluorescence signals. After the measurement point was selected in x and y directions, the precise focusing of the excitation light was done by moving the head in z direction, followed by the registration of the fluorescence signals. An acquisition time of 1 s was set to register both, the short-wavelength and long-wavelength fluorescence light. The laser power in the focus was set to 1.8 mW at *λ*_1_ = 532 nm and to 90 mW at *λ*_2_ = 785 nm. The registered fluorescence spectra were submitted to evaluation without further pre-processing.

### Transmission spectroscopy and fluorescence spectroscopy of hemoglobin and protoporphyrin IX

Transmission spectra of human hemoglobin (CAS 9008–02-0, Sigma-Aldrich, St. Louis, USA) solved in isotonic sodium chloride solution 0.9%, and synthetic protoporphyrin IX (CAS 553–12-8, Medchem Express Fisher Scientific, Waltham, USA) solved in denatured ethanol were recorded in a 1-cm cuvette using a Cary 5 spectrometer (Agilent, Santa Clara, USA; 200–800 nm) and a near-infrared (NIR) spectrometer (Vertex 70, Bruker GmbH, Ettlingen, Germany; 800–1000 nm). The concentration of the hemoglobin solution was 7 µmol/l and that of the protoporphyrin IX solution was 7.8 µmol/l. Background spectra of the pure solvent were registered under the same conditions. It should be noted that human hemoglobin was used because this is available in pure form. The in ovo sampling of embryonic blood is difficult and a proven protocol for separation and purification of hemoglobin is not available. Furthermore, the fluorescence and absorption characteristics of hemoglobin are determined to a large extent by the heme group.

Fluorescence spectra of the compounds were recorded with the in ovo optical set up and measuring head. Both samples were measured in a lockable cuvette with a volume of 3500 µl. The concentrations of the solutions were the same as for the transmission measurements. The integration time for both spectral regions was 2 ms. All other parameters were identical to those of the in ovo measurements (see above).

## Results and discussion

**Fig. 3:**
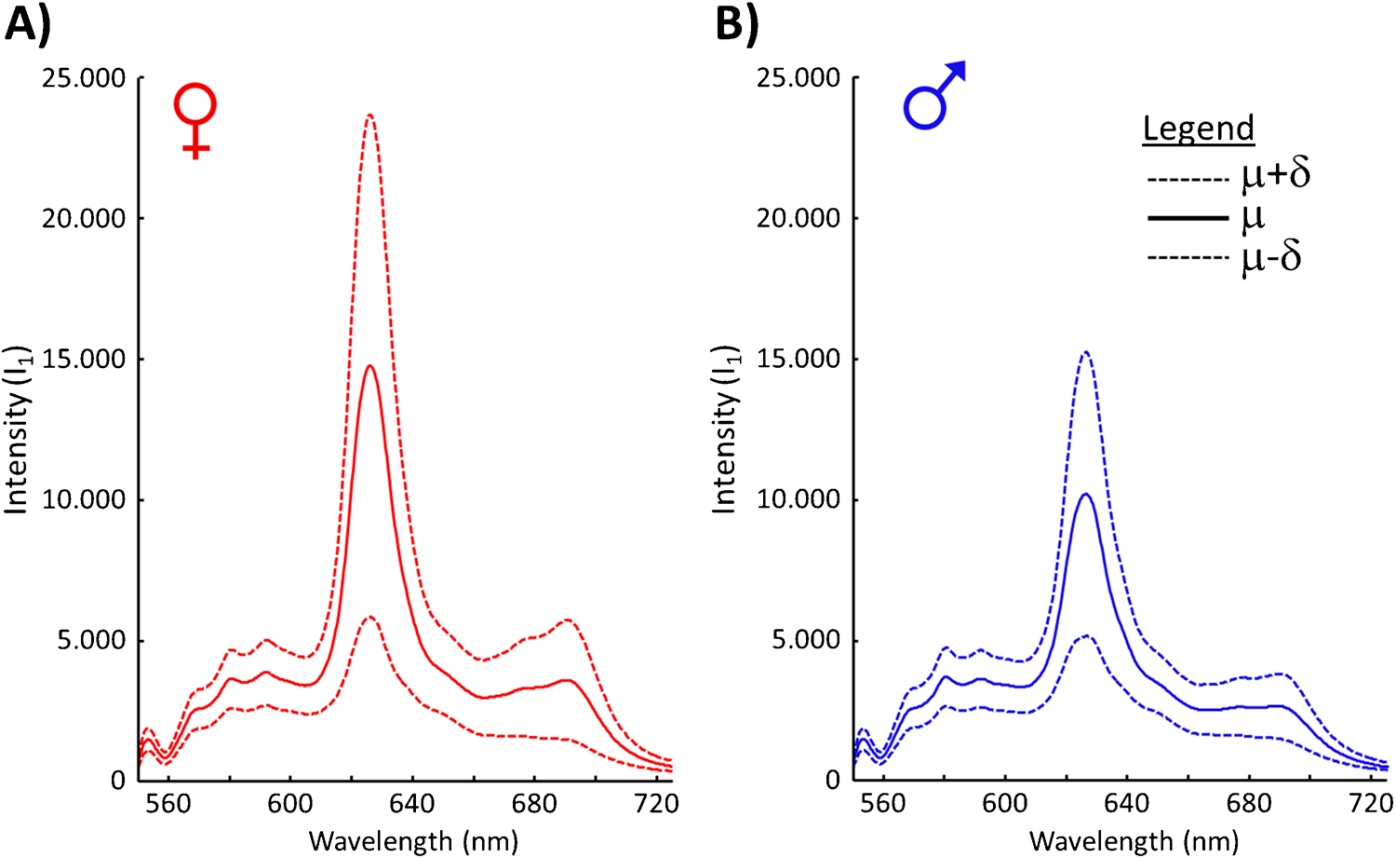
Calculated mean spectra (*μ*) and standard deviation (*δ*) of in ovo fluorescence (*I*_1_) recorded at an excitation of *λ*_1_ = 532 nm for (**A**) female embryos (red) and (**B**) male embryos (blue)

Soon after the incubation begins, the yolk moves from the center of the egg to the upper end. Embryonic blood vessels are forming beneath the yolk membrane. Similar as described in [14], the extraembryonic blood can be analyzed in ovo by fluorescence spectroscopy. In order to get a first overview of the registered in ovo fluorescence spectra, the mean spectrum and standard deviation were calculated from all spectra of female and male embryos, respectively. The assignment of spectra to the female or male group was done according to the result of feather sexing. Figure 3 shows the fluorescence profiles at an excitation of *λ*_1_ = 532 nm.

**Fig. 4:**
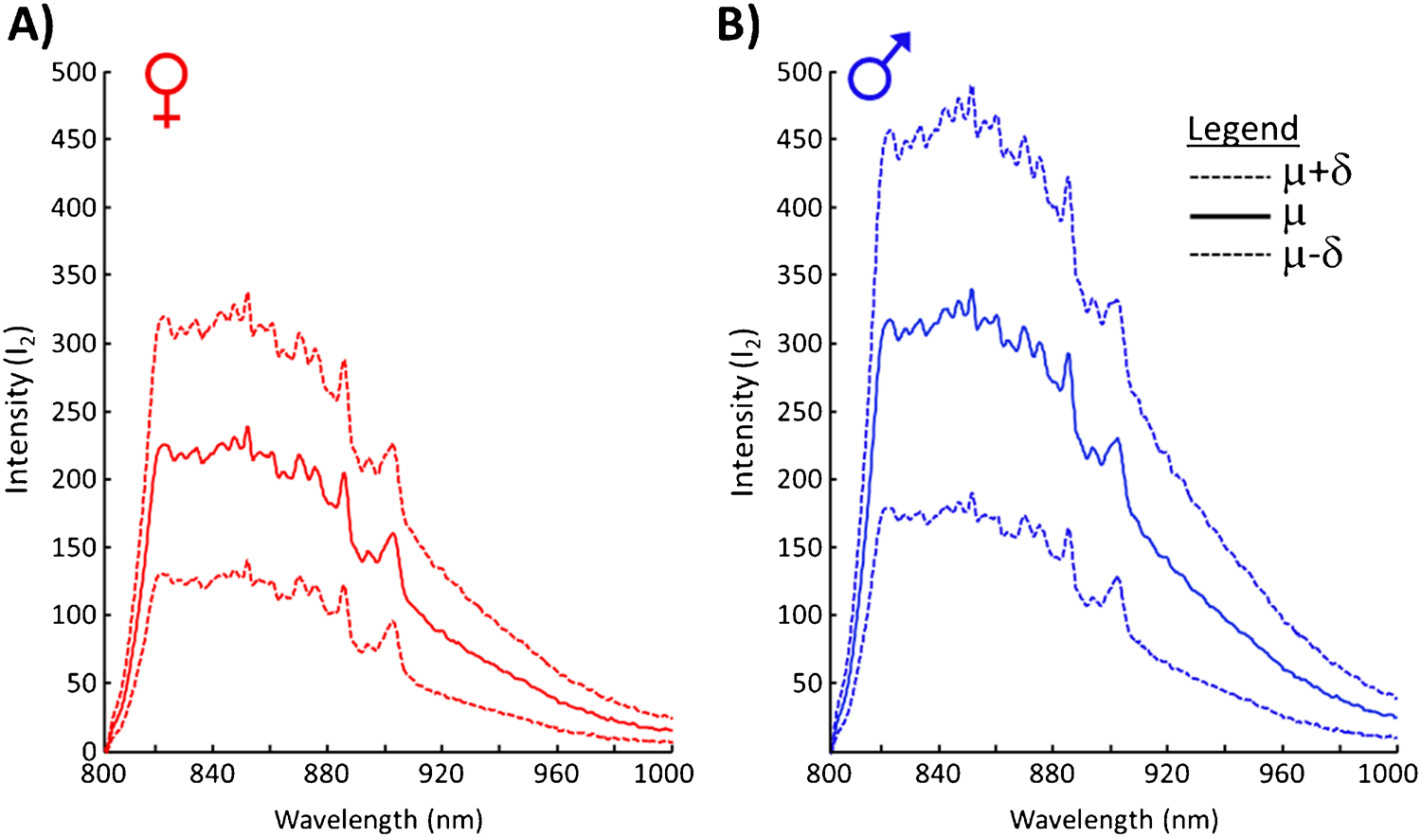
Calculated mean spectra (*μ*) and standard deviation (*δ*) of in ovo fluorescence (*I*_2_) recorded at an excitation of *λ*_2_ = 785 nm for (**A**) female embryos (red) and (**B**) male embryos (blue)

All fluorescence spectra show a maximum intensity around 628 nm. While the spectral profile of female and male embryonic blood is similar, it is apparent that blood of female embryos shows in this spectral range in average a stronger fluorescence intensity than blood of male embryos. Although the different intensities suggest a potential discrimination between female and male embryos taken into consideration the standard deviation, the sex assignment based on this fluorescence signals alone would become unreliable. Figure 4 shows, similar to Fig. 3, the calculated mean spectra and standard deviation of fluorescence spectra at an excitation of λ_2_ = 785 nm.

Here, the blood of male embryos shows in average a stronger fluorescence intensity than the blood of female embryos. In comparison to the fluorescence signals of an excitation wavelength *λ*_1_ = 532 nm, the fluorescence signals for an excitation of *λ*_2_ = 785 nm are weaker and broader. Individual Raman bands of the inner eggshell membrane are observable. The strong slope between 800 and 820 nm arises from the transmission characteristic of an optical filter blocking the excitation laser line of *λ*_2_ = 785 nm.

**Fig. 5:**
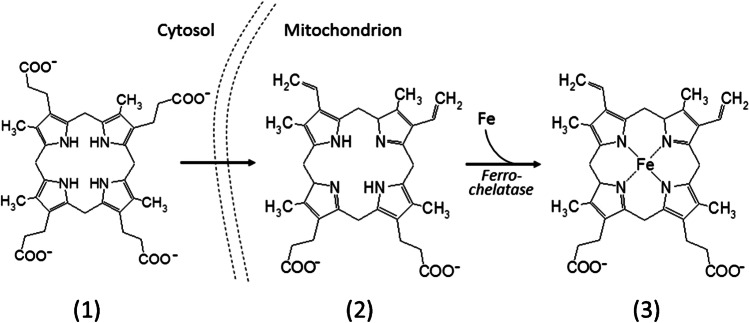
Illustration final steps of the heme synthesis pathway from coproporphyrinogen (**1**) to protoporphyrin IX (**2**) and the terminal step catalyzed by ferrochelatase to the heme (**3**)

From the spectra, the question arises which blood constituents are responsible for fluorescence between 560 and 720 nm and between 800 and 1000 nm. One possibility is that constituents of the heme synthesis are at least partly responsible for these signals. Within the complex pathway of heme synthesis, there are two molecules that show a substantial fluorescence. Figure 5 shows a section of the heme synthesis pathway [26]. Briefly, heme synthesis starts in mitochondria and intermediates enter the cytosol, where coproporphyrinogen (**1**) is generated. After transport back to mitochondria, coproporphyrinogen decarboxylates and oxidizes two propionic side chains to vinyl groups. Protoporphyrinogen is formed and further oxidized to protoporphyrin IX (**2**). Finally, the enzyme ferrochelatase catalyzes the formation of heme (**3**) by inserting Fe^2+^ into protoporphyrin IX.

**Fig. 6:**
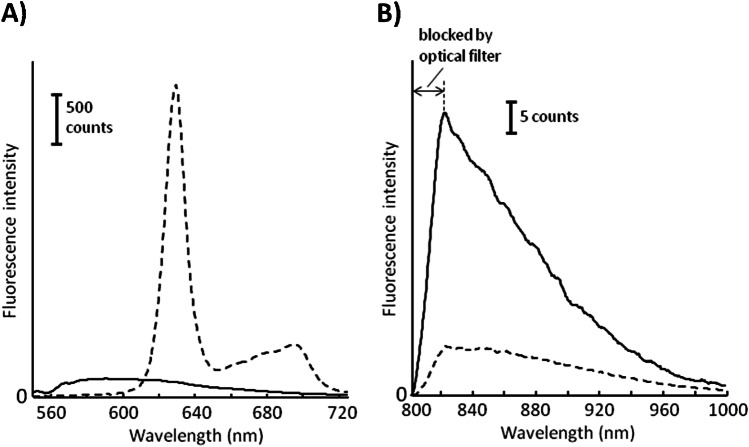
Fluorescence spectra of protoporphyrin IX (dashed) and human hemoglobin (bold) for excitation wavelengths (**A**) *λ*_1_ = 532 nm and (**B**) *λ*_2_ = 785 nm. Note, for better illustration, both fluorescence spectra of hemoglobin are multiplied by a factor of 5

Coproporphyrinogen (**1**) is a high transparent component showing no fluorescence. Protoporphyrin IX (**2**) exhibits a comparatively strong fluorescence in the red and NIR while heme (**3**) shows only a very weak fluorescence in these spectral regions. The fluorescence spectra of synthetic protoporphyrin IX and human hemoglobin are plotted in Fig. 6.

**Fig. 7:**
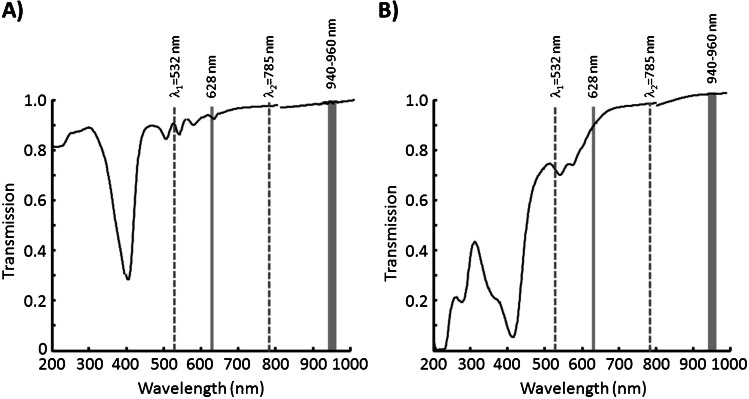
Transmission spectra of (**A**) synthetic protoporphyrin IX and (**B**) of human hemoglobin. The dotted gray lines indicate the excitations laser lines. Gray bars represent spectral regions used for evaluation in ovo fluorescence intensities

The spectra shown in Fig. 6A and B were registered with the in ovo measuring head (see Fig. 2). Both samples were placed in cuvettes with 10-mm path length. However, the effective path length is determined by the focus of the excitation lasers. The spectral fluorescence characteristic of protoporphyrin IX is similar to the measured in ovo fluorescence (c.f. Figure 3). In contrast to protoporphyrin IX, hemoglobin shows only very weak fluorescence in the region between 560 and 720 nm. However, it is known that concentration of hemoglobin in human erythrocytes is much higher than concentration of protoporphyrin IX. Thus, it can be assumed that in the egg, the concentration of embryonic hemoglobin is also higher than that of the precursor protoporphyrin IX so that especially between 800 and 1000 nm, hemoglobin does predominantly contribute to the fluorescence measured in ovo. Hemoglobin as the main source of the observed NIR fluorescence was also indicated by comparing the time courses of in ovo fluorescence intensity and hemoglobin content during early erythropoiesis [12]. The spectral profile in the range between 800 and 1000 nm also corresponds well with the in ovo fluorescence spectra shown in Fig. 4. However, the fluorescence of protoporphyrin IX (560–720 nm) and hemoglobin (800–1000 nm) alone does not explain the sex-specific differences of the observed in ovo fluorescence intensities *I*_1_ and *I*_2_ (c.f. Figure 3 and Fig. 4). To understand the different fluorescence intensities, the transmission of protoporphyrin IX and hemoglobin has to be considered. The transmission spectra of both components are plotted in Fig. 7.

The transmission spectrum of protoporphyrin IX (Fig. 7A) is characterized by the strong Soret band at 408 nm and the four Q-bands between 450 and 650 nm. The Soret band of hemoglobin (Fig. 7B) is also located around 408 nm. The Q-bands are merged to broad absorption band between 500 and 650 nm. The transmission spectra reveal that hemoglobin absorbs stronger the light in the green than protoporphyrin IX. This applies to both the excitation line (*λ*_1_ = 532 nm) and the fluorescence light at the maximum of the protoporphyrin IX fluorescence around 628 nm.

Table 2 lists the measured transmission values as well as the calculated extinction coefficients of both compounds for the spectral positions and ranges used for the in ovo fluorescence spectroscopy. In particular, hemoglobin has at *λ*_1_ = 532 nm a high extinction coefficient. In contrast to the green spectral range, the absorption of protoporphyrin IX and hemoglobin is only very weak in the range between 940 and 960 nm.

**Table 2:** Measured transmission (*T*) and calculated extinction coefficients (*ε*) of synthetic protoporphyrin IX and human hemoglobin at the spectral positions (*λ*) used for in ovo fluorescence spectroscopy

	Synthetic protoporphyrin IX		Human hemoglobin	
*λ* (nm)	*T* (%)	*ε* (l mol^−1^ cm^−1^)	*T* (%)	*ε* (l mol^−1^ cm^−1^)
532	87.3	19,417	69.3	47,007
628	92.3	11,467	86.8	18,168
785	97.3	3857	95.8	5481
940	98.8	1774	99.4	714
960	98.9	1538	99.5	594

Consequently, the concentration of hemoglobin in the embryonic erythrocytes modulates the fluorescence intensities of protoporphyrin IX. Male chicken embryos have, in contrast to female chicken embryos, obviously at the early incubation days a higher regulated metabolism accompanied by a higher content of hemoglobin. It was recently published that ferrochelatase of the domestic chicken (*Gallus gallus f. dom.*) is encoded by a single nuclear gene located at the gender specific sex chromosome Z [27]. Birds have no dosage compensation system for the Z chromosome similar to the X inactivation observed in mammals [28]; higher expression levels of Z-linked genes in homozygote male (ZZ) than in hemizygote female (ZW) chicken embryos are the result [29]. Therefore, it can also be postulated that male chicken embryos produce more heme and finally also more hemoglobin than female chicken embryos. This hypothesis is supported by the study of Rahman et al. which have investigated the spectral transmission of eggs at day 3 [30]. The authors found that eggs of male embryos show a higher absorbance of hemoglobin than eggs of female embryos, indicating a faster erythropoiesis of male embryos in the first phase of incubation. Also Tagirov et al. found in a study of sexual dimorphism of the early embryogenesis that male chicken embryos grow faster than females [31]. Thus, it can be assumed that the ratio of hemoglobin to protoporphyrin IX increases with the development of the embryo.

Although not all mechanisms and their interrelationships are yet understood, it can be hypothesized that the observed sex-related differences in the fluorescence intensities are based on the embryonic heme synthesis.

Prediction of sex from spectra registered in ovo can be done using supervised classification. Methods for this have already been successfully applied in previous work [11–13]. Due to the complementarity between the signals of the two spectral regions, a more simple classification approach can now be performed. For the in ovo sexing, the fluorescence signals of excitation *λ*_1_ = 532 nm and *λ*_2_ = 785 nm are ratioed according to

1 $$F=\frac{{\int }_{940}^{960}{I}_{2}\left(\lambda \right) d\lambda }{\mathrm{max}({I}_{1}(620\dots 640 \mathrm{nm}))}$$

**Fig. 8:**
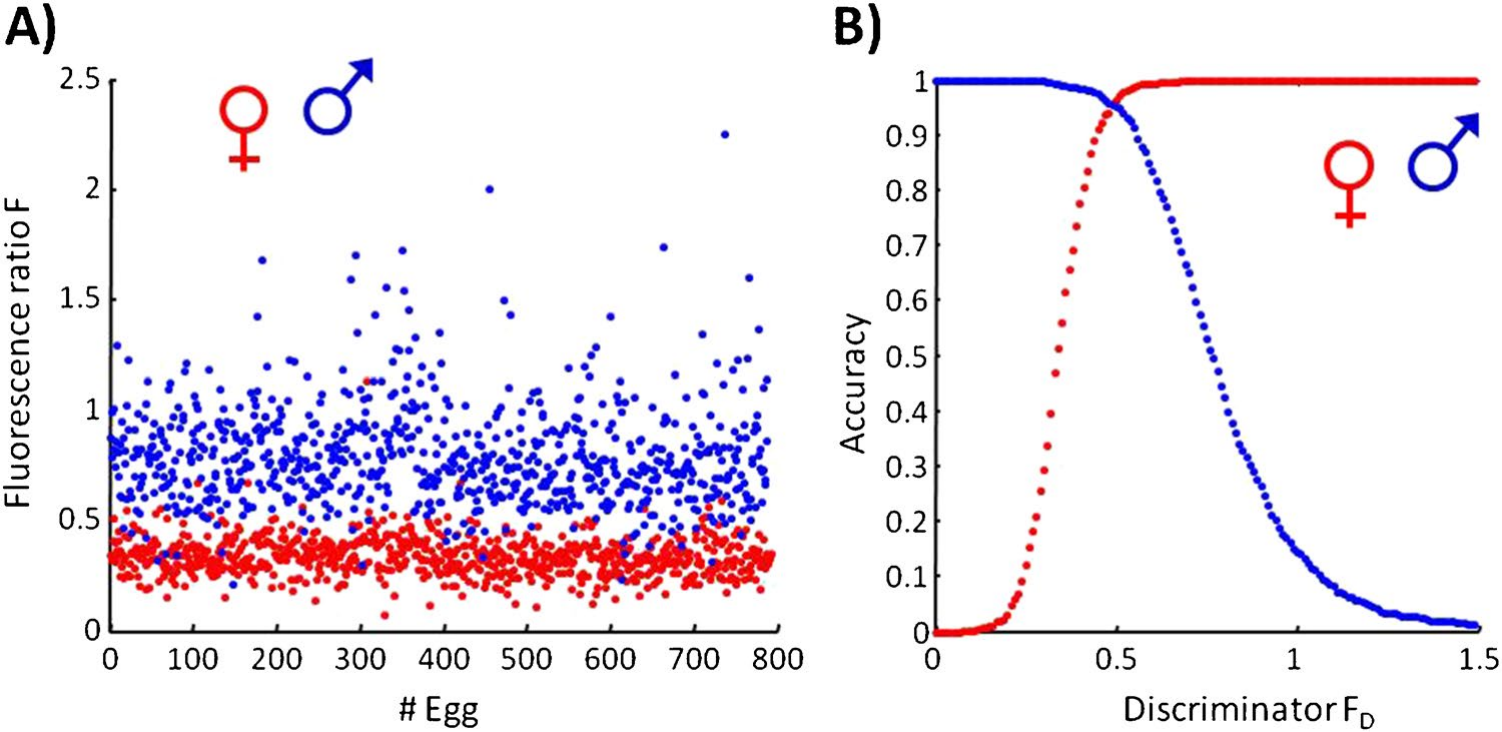
(**A**) Scatterplot of the calculated fluorescence ratio F (see Eq. (1)) for all in ovo sexed eggs. (**B**) Accuracy versus discriminator *F*_D_. Assignment of F to female (red dots) or to male (blue dots) embryos is based on feather sexing

The scatter plot of the fluorescence ratio F for female and male embryos is presented in Fig. 8A. Even at first glance, it is clear that female and male embryos can be very easily distinguished. For classification, a discriminator *F*_D_, i.e., a threshold, can be introduced. It is obvious that the choice of the threshold determines the accuracy for one or the other sex in opposite direction. Figure 8B shows the accuracy for female and male embryos as a function of the discriminator *F*_D_. The accuracy is defined as the proportion of correct determined embryos to the total number of hatched chicks for the female and male groups, respectively. Eggs that were in ovo sexed but from which no chick hatched were not included in the calculation.

The intersection of the curves is approximately at a discriminator value of *F*_D_ = 0.49 with a corresponding accuracy of 96% for both sexes. Classification of the in ovo spectra according to Eq. (1) and at a threshold of F_D_ = 0.49 resulted in 795 female embryos and 786 male embryos. For female embryos, there were 759 matches and for male embryos, 752 matches between in ovo and feather sexing. The results show that with the analytical techniques of fluorescence spectroscopy, the sex-relevant molecular information can be registered in ovo. At the same time, fluorescence spectroscopy as a non-contact technique with respect to the living embryo ensures a high hatching rate of normally developed and healthy chicks.

The prediction of sex by in ovo spectroscopy and agreements and discrepancies to the subsequent feather sexing are listed in Table 3.

**Table 3:** Summarizing the results of spectroscopic in ovo sexing and feather sexing of hatched chicks

Hatched chicks of in ovo sexed eggs	Prediction of the sex by in ovo spectroscopy (*F*_D_ = 0.49)	Feather sexing of hatched chicks		Accuracy
		Consistent with in ovo spectroscopy	Different to in ovo spectroscopy	
1581	Female: 795	759	36	0.96
	Male: 786	752	34	0.96

The reasons for misclassified embryos are not yet understood. As in all previous studies [11–14], similar deviations were observed too; it can be assumed that variations in the embryonic developmental stage or in the general biochemical profile are reasons for the misclassification. Furthermore, a closer look at the scatter plot also reveals that the in ovo signal F of a few female as well as male chicks is clearly emerged in the other, “wrong” sex group. The reasons for this clear discrepancy are not known. Measurement errors can be excluded after a detailed analysis of measurement procedure and data. As all misclassified chicks have been raised, a possible inherent sexual identity based on genuine male to female chimaeras [32] could not be found in any of the animals so far. It might be that there are discrepancies between the biochemical and genetic sex which are not yet understood.

In addition to the accuracy of the in ovo sexing approach, the hatching rate is also an important parameter of the method. For the evaluation of the hatching rate, a similarly large number of eggs were hatched with complete preservation of their native state. The hatching results of this control group and of in ovo sexed eggs are summarized in Table 4.

**Table 4:** Total numbers of eggs and hatched chicks of the ovo sexing and control group

Group	Eggs with vital embryos at d4	Death between d4 and d18	Dead-in-shell after d18	Hatched chicks	Hatching rate
In ovo sexed	1641	31	29	1581	96.3%
Control	1749	33	35	1681	96.1%

For both groups, the hatching rate is nearly identical. In other words, opening and spectroscopic in ovo sexing do not lead a priori to a reduced hatching rate.

It should be noted that the study was performed as a blind test. The sex of each egg was documented prior to hatching and then compared to the result of feather-sexing. The feather sexing as well as the comparison to the predicted sex was performed by independent experienced specialists from the FLI and the Agri-Advanced Technologies GmbH (Visbeck, Germany).

The here described and patented technique of the two-wavelength fluorescence spectroscopy [33] allows an early in ovo sex determination. Due to its applicability at an early stage combined with a high accuracy and especially because of the non-decreased hatching rate, it is well suited for a practical application in hatcheries in order to overcome the killing of day-old male chicks and to solve a current animal welfare problem.

At this point, it is stated that the methodology described here has been filed as a patent and the granting of the property right is in prospect.

## Conclusions

Fluorescence spectroscopic in ovo sexing demonstrates how analytical chemistry can contribute to solving one of the current major problems of animal welfare. As a non-contact method, it ensures a high hatching rate of the chicks. The dual in ovo fluorescence excitation at 532 nm and 785 nm identifies the sex of the embryo after a few days of incubation. Based on a sample size of more than 1600 eggs, an accuracy of 96% could be determined for both sexes. While at 628 nm predominantly fluorescence of protoporphyrin IX, a precursor to hemoglobin, in the extraembryonic blood is detected, in the range between 800 and 1000 nm protoporphyrin IX and hemoglobin contribute to the fluorescence signal. The sex-specific difference in fluorescence intensities at the excitation wavelengths is caused by the absorption of the heme group. The blood of female embryos shows in average a higher fluorescence intensity at 628 nm than the blood of male embryos. The blood of male embryos shows in average a higher fluorescence intensity than the blood of female embryos in the range between 800 and 1000 nm. This is due to a generally increased heme synthesis of male embryos. The hatching rate of eggs analyzed in ovo is identical to the hatching rate of eggs not examined.
